# Infective Endocarditis and Septic Emboli: Diagnostic Delays, Implications, and Challenges

**DOI:** 10.7759/cureus.83117

**Published:** 2025-04-28

**Authors:** Ali M Ibnian, Maria Elizabeth Vincent, Jovita Amadi

**Affiliations:** 1 Internal Medicine, Milton Keynes University Hospital NHS Foundation Trust, Milton Keynes, GBR

**Keywords:** austrian syndrome, cardiac vegetations, cerebral septic emboli, infective endocarditis, osler's triad, streptococcus pneumoniae infective endocarditis

## Abstract

Infective endocarditis (IE) presents significant diagnostic challenges and needs a high index of clinical suspicion. This study describes a case illustrating these complexities. A 69-year-old woman initially presented with confusion and expressive dysphasia, following five days of treatment for left-sided otitis media. Further investigations revealed evidence of Streptococcus infection. The patient was diagnosed with pneumococcal infective endocarditis, complicated by intracranial septic emboli and multiorgan failure. This resulted in prolonged hospital stay and the development of multi-morbidity and increased frailty index.

IE was diagnosed only upon readmission, leading to delays in treatment. This study underscores the importance of early suspicion and repeat investigations in IE. Evolving diagnostic criteria emphasize a multidisciplinary approach to managing these life-threatening conditions.

## Introduction

Infective endocarditis (IE) is an infection affecting the heart endothelium, mainly the heart valves. It poses a high mortality rate, reaching up to one-third of the affected patients within the first month of diagnosis. Early disease recognition and proper early treatment are crucial to prevent mortality and morbidity [1,2].

Diagnosing IE is a formidable challenge and requires a high index of clinical suspicion. Diagnostic delays are frequently associated with severe complications, invasive surgical interventions, and poor patient outcomes [1,3]. Diagnostic criteria for IE have evolved over time, with the latest revisions reflected in the Duke’s criteria, which incorporate advances in radiological and microbiological diagnostic tools [4]. These advances have increased the ability to diagnose IE early, but significant barriers to timely diagnosis remain.

Septic embolic events are prevalent; they occur in approximately a quarter of patients diagnosed with infective endocarditis and are associated with a higher mortality rate. Though still underestimated, a higher clinical suspicion of an infective cause in general, and infective endocarditis in particular, should be considered when septic emboli are identified on brain imaging [2,5]. We report a case affected by diagnostic delays contributed by the presence of septic emboli. The main missing clue for diagnosis was the presence of cerebral septic emboli.

*Streptococcus pneumoniae *infection may rarely present as a combination of meningitis, pneumonia, and endocarditis - an entity previously described as Austrian syndrome or Osler’s triad - which causes significant morbidity and high mortality, even with appropriate medical and surgical treatment [6]. The present case describes a variant of the same, if we take a broader view of a multi-systemic infectious process, including septic brain emboli, otitis media with mastoiditis, and endocarditis, which, to a lesser extent, correspond to the components of Osler's triad, respectively.

## Case presentation

A 69-year-old woman with an unremarkable medical background presented in septic shock, a one-week history of progressive otalgia with intermittent fever, and left inflamed mastoid. Otoscopy showed a bulging tympanic membrane, and the CT head confirmed mastoiditis.

**Figure 1:**
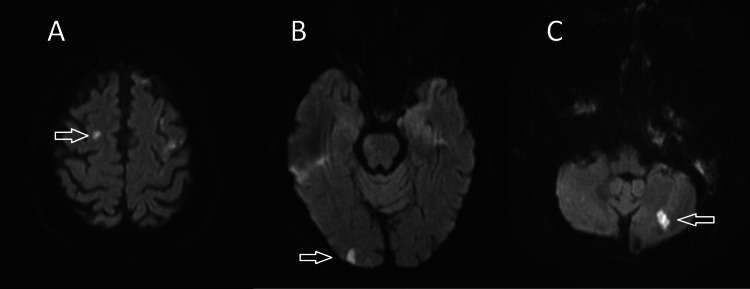
MRI head showing evidence of multiple small acute infarcts involving (A) right frontal lobe, (B) right occipital lobe, and (c) left cerebellar hemisphere (arrows).

On the next day of admission, she developed transient expressive aphasia, without other neurological symptoms. Examination was unremarkable, barring an incidental finding of a diastolic aortic murmur. MRI head showed evidence of multiple acute microinfarcts involving both frontal lobes, right occipital lobe, and left cerebellar hemisphere (Figures 1A-1C).

An inpatient transthoracic echocardiogram (TTE) revealed aortic masses and prolapse of the right coronary cusp of the aortic valve, along with a normal ejection fraction (EF%) and preserved cardiac wall motion. Unfortunately, the echocardiogram images were unavailable and could not be included in this article. The patient showed clinical improvement after five days of intravenous ceftriaxone and was discharged with plans for an outpatient transesophageal echocardiogram (TEE) for further evaluation.

**Figure 2:**
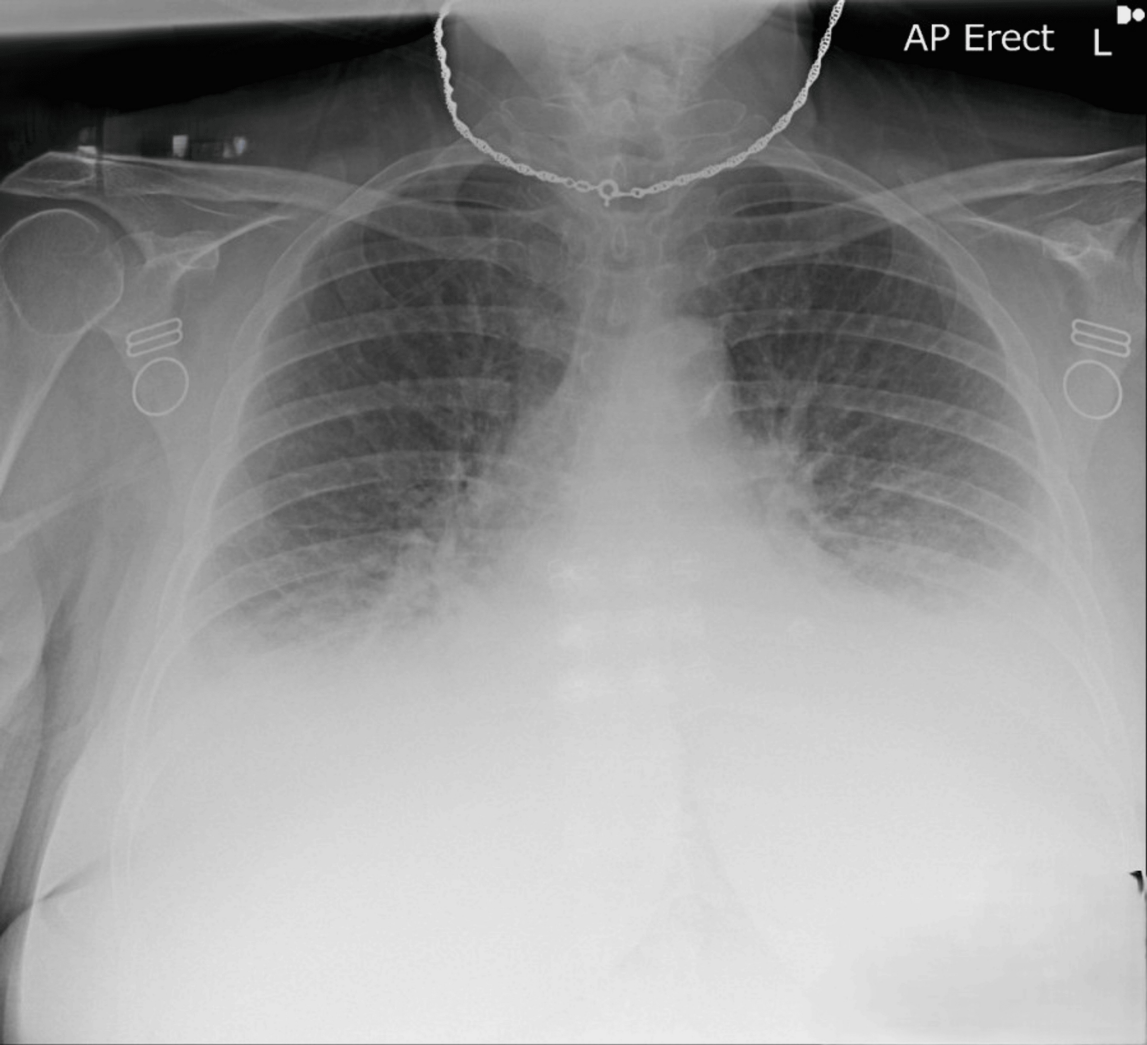
Chest X-ray showed features of heart failure, including bilateral pleural effusion, congested hila, and pulmonary edema.

**Figure 3:**
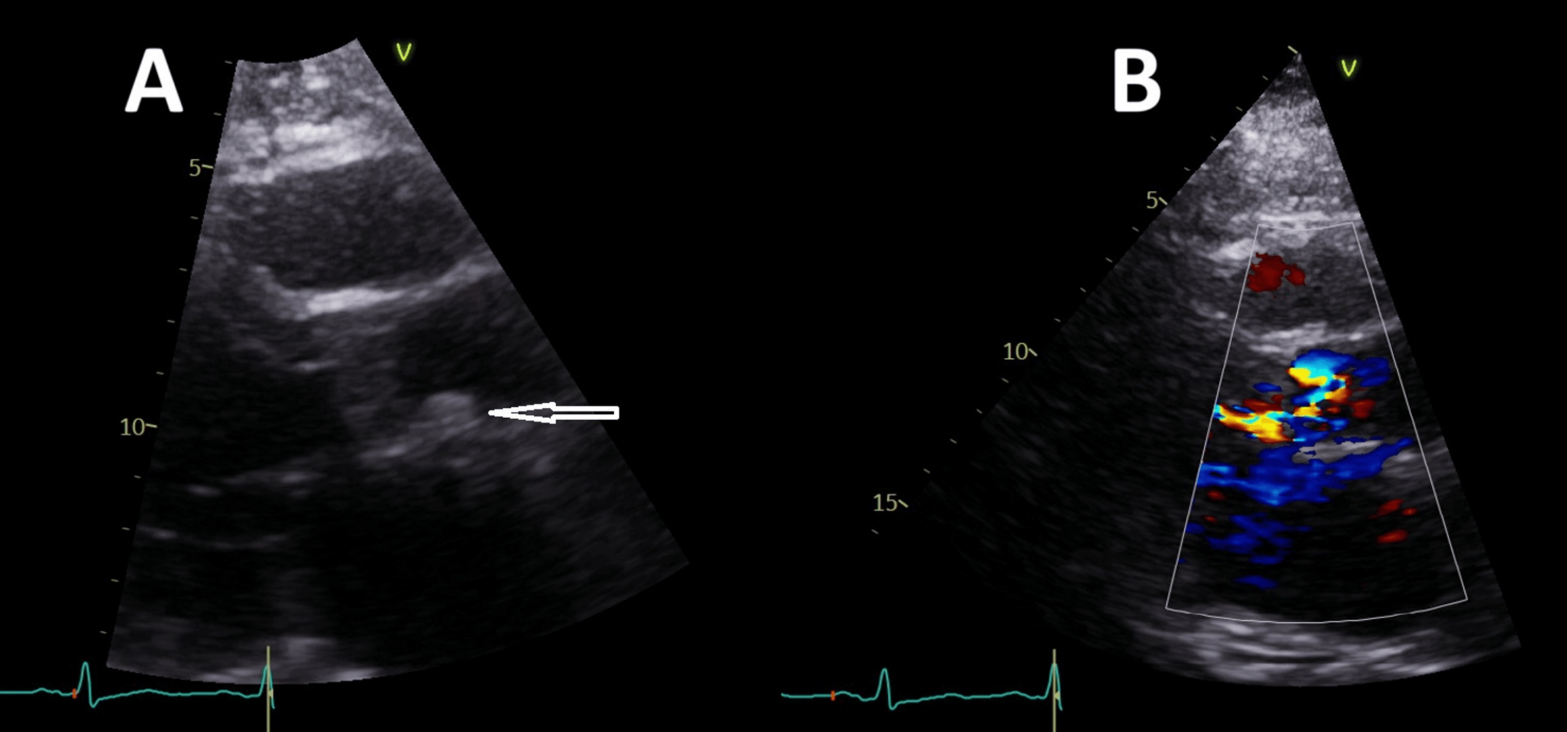
TTE showing (A) aortic valve vegetations (arrow) and (B) an aortic regurgitation jet. TTE: transthoracic echocardiogram

One week later, she presented again with progressive dyspnea. Physical examination and chest X-ray were suggestive of fluid overload, along with the absence of a silent diastolic murmur, which suggests the presence of a diastolic aortic murmur (Figure 2). A repeat TTE revealed moderate reduction in EF% with enlargement of the aortic masses and new masses on the mitral valve described as vegetations; the aortic vegetation and the regurgitation jet are shown in Figures 3A, 3B.

There was a diagnostic conundrum, as differentials for her presentation included atrial myxoma and non-bacterial endocarditis, thereby warranting further investigation and contributing to complications in the form of heart failure.

Unfortunately, she continued to deteriorate despite medical management, necessitating a surgical approach in the form of valvular replacement. Aortic valve biopsy 16S rRNA PCR was positive for *Streptococcus pneumoniae*. Subsequently, she completed six weeks of intravenous antibiotics with a good recovery thereafter.

## Discussion

The initial diagnostic challenge was recognizing the clinical significance of septic emboli and correlating it with the transthoracic echocardiogram (TTE) abnormalities, specifically, the "valvular masses." Vegetation is defined as an echodense, mobile mass tethered to a native or prosthetic heart valve, cardiac mural endocardium, or intracardiac implanted prosthesis, located in the trajectory of a regurgitant jet, in the absence of an alternative anatomical explanation [7,8]. In the present case, although the description matched the definition of vegetation, the inconsistent terminology "masses" was used in the initial TTE report, and therefore, it was not recognized as a sign of infective endocarditis (IE). Valve vegetation is an important clue for IE diagnosis. However, echocardiography still has significant limitations, and novel imaging techniques are increasingly being exploited to improve diagnostic potential [4,9]. 

Infectious cause, in particular infective endocarditis, should be considered when both embolic event and febrile illness are present simultaneously. Increasing awareness regarding this point is crucial. In the reported case, signs of septic emboli were present, but it did not initially lead the treating teams to the diagnosis of IE. One of the challenges in the present case was determining whether the number of positive cultures met the major criterion for IE.

The primary cause of septic emboli was mastoiditis. ENT causes of IE are rare. We are aware of one previously published case report that demonstrated mastoiditis can directly cause endocarditis [10]. Establishing an early diagnosis, involving a dedicated IE team early, and performing prompt surgical intervention when indicated are established measures that improve patient outcomes. Early surgical intervention, with less than seven days of pre-operative antibiotic therapy, is associated with a lower risk of mortality compared to surgery performed between eight and 20 days after the initiation of antibiotics [11].

## Conclusions

*Streptococcus pneumoniae* is one of the common bacterial pathogens causing infection in the respiratory tract, endocardium, and brain meninges. The presence of infection in all three sites is a fatal condition known as Austrian syndrome, a definition that might apply to the present case. Although the causal relationship between mastoiditis and infective endocarditis requires further study and investigation, this report may provide evidence suggesting a potential link that warrants deeper exploration.

A high index of suspicion, greater attention to physical examination findings, and repeat echocardiography, preferably transesophageal, should be prioritized when the initial TTE is negative or inconclusive, especially in the presence of microinfarcts, fever, and an incidental murmur, to further investigate for endocarditis and ensure a timely and accurate diagnosis.
